# Effect of childhood trauma on patients with schizophrenia

**DOI:** 10.1192/j.eurpsy.2021.1462

**Published:** 2021-08-13

**Authors:** R. Ouali, R. Sellami, N. Cheffi, S. Elleuch, F. Cherif, R. Masmoudi, J. Masmoudi

**Affiliations:** Psychiatrie “a” Department, Hedi Chaker Hospital University -Sfax - Tunisia, sfax, Tunisia

**Keywords:** childhood, trauma, schizophrénia

## Abstract

**Introduction:**

Recent research shows that exposure to trauma, such as child abuse, may result in a heightened risk of developing schizophrenia and worsening of positive symptoms in schizophrenic patient.

**Objectives:**

The objective of this study was to examine the relation between childhood abuse and psychotic symptoms in patients with schizophrenia.

**Methods:**

Participants were outpatients of Hedi chaker University Hospital Center in sfax, Tunisia, recruited between January and July of 2019, diagnosed with schizophrenia or schizoaffective disorder. The Childhood Trauma Questionnaire (CTQ-SF), the Positive and Negative Syndrome Scale (PANSS) were administered in this study to evaluate respectively childhood trauma and psychotic symptoms

**Results:**

44 patients were included in this study with an average age 39,81 ±9,7. The rate of emotional abuse was 15.9%, physical abuse 31.8%, sexual abuse 15.8%, emotional neglect 6.8% and physical neglect 18.2%. PANS positive score (r=0,59 ; p< 10^-3^ ), PANS negative score (r=0,55 ; p< 10^-3^ ) and PANS psychopathology score(r =0,45, p<0,002) were higher in patients who had a history of childhood trauma in comparison with those who did not report experiencing this.

**Conclusions:**

This study confirms that a history of Childhood trauma may have a serious impact in patients with schizophrenia.

